# Supplementary material for: Spatial and single-cell transcriptomics capture two distinct cell states in soybean defense response to Phakopsora pachyrhizi infection
Source: Front Plant Sci. 2025 Sep 11;16:1637176. doi: 10.3389/fpls.2025.1637176 (PMC12460401; doi:10.3389/fpls.2025.1637176)
Supplement: Supplementary file 1 [file DataSheet1.pdf]

Supplementary Material

Figure S1

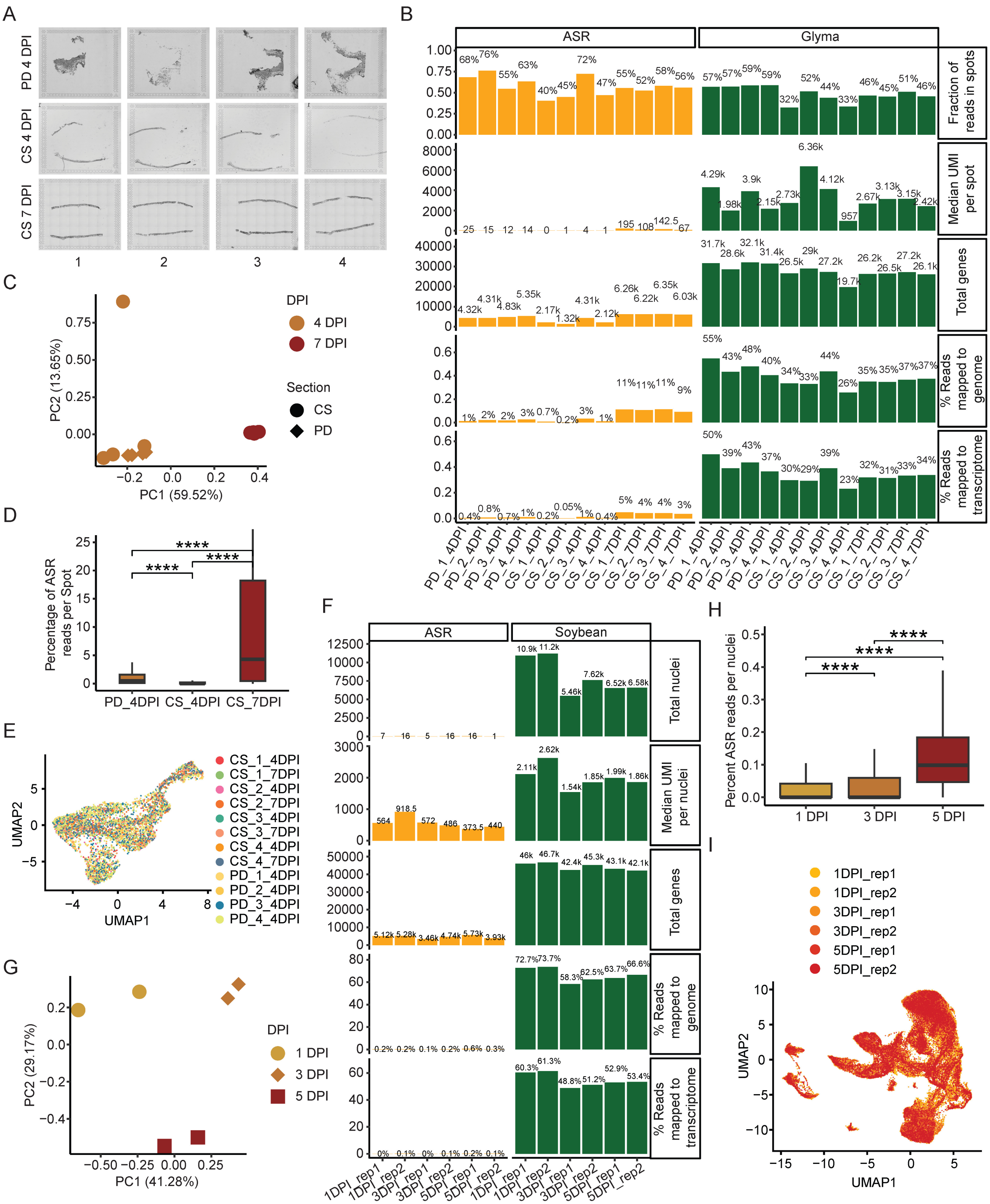

Supplemental Figure 1. A) Brightfield uncropped images of leaf sections used for spatial analysis. **B)** Sequencing metrics for spatial analysis separated by genome. **C)** PCA of pseudo-bulked normalized counts for spatial analysis. **D)** Percent ASR reads per spot measured across all spatial transcriptomics samples. * indicates $p < 0.05$, ** indicates $p > 0.01$, *** indicates $p < 0.001$, **** indicates $p < 0.0001$. **E)** UMAP of all spots from all sections. **F)** Sequencing metrics for snRNA-seq separated by genome. **G)** PCA of pseudo-bulked counts for snRNA-seq. **H)** Percent ASR reads per nuclei measured across days of ASR infection. * indicates $p < 0.05$, ** indicates $p > 0.01$, *** indicates $p < 0.001$, **** indicates $p < 0.0001$. **I)** UMAP of all nuclei from all snRNA-seq samples.

Figure S2

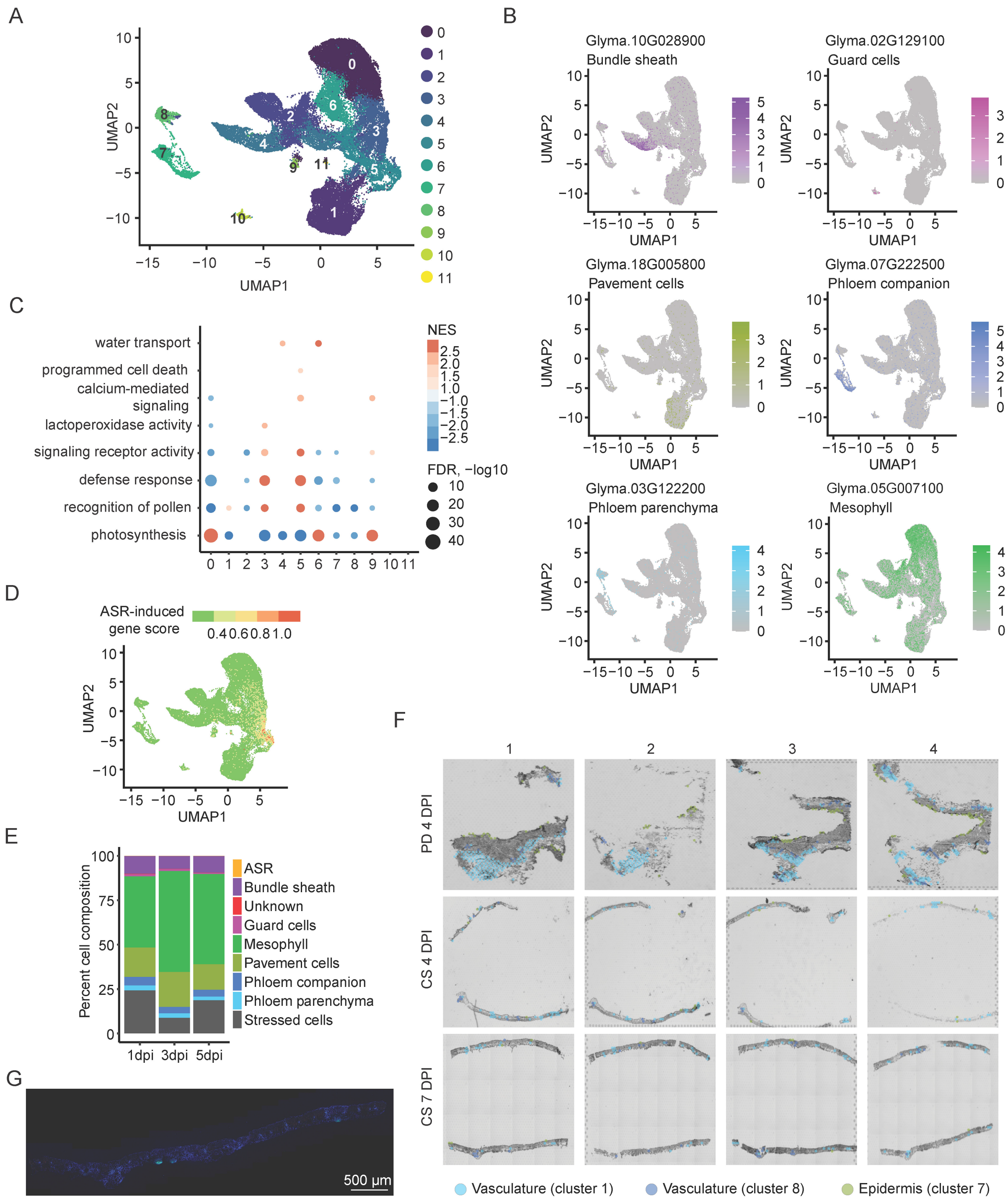

Supplemental Figure 2. A) UMAP of snRNA clusters. **B)** Expression of known leaf cell type marker genes in snRNA-seq dataset. **C)** Dot plot of enriched GO terms associated with each snRNA-seq cluster's marker genes. Size of dots corresponds to -log10 of adjusted *p* value, colored by Normalized Enrichment Score (NES). **D)** ASR-induced gene expression described as a single score across snRNA UMAP. **E)** Cell type composition in snRNA across infection time. **F)** Spatial clusters 1 and 8 (blue) overlapping with midrib vascular bundles in cross section as well as visible veins in paradermal sections. Spatial cluster 7 (green) outlines paradermal sections and few external spots in

cross sections. G) Fluorescence image of 7 DPI cross section. Green fluorescence shows localization of fungal cells stained with wheat germ agglutinin Alexa Fluor 488 conjugate. Scale bar is 500 µm.

Figure S3

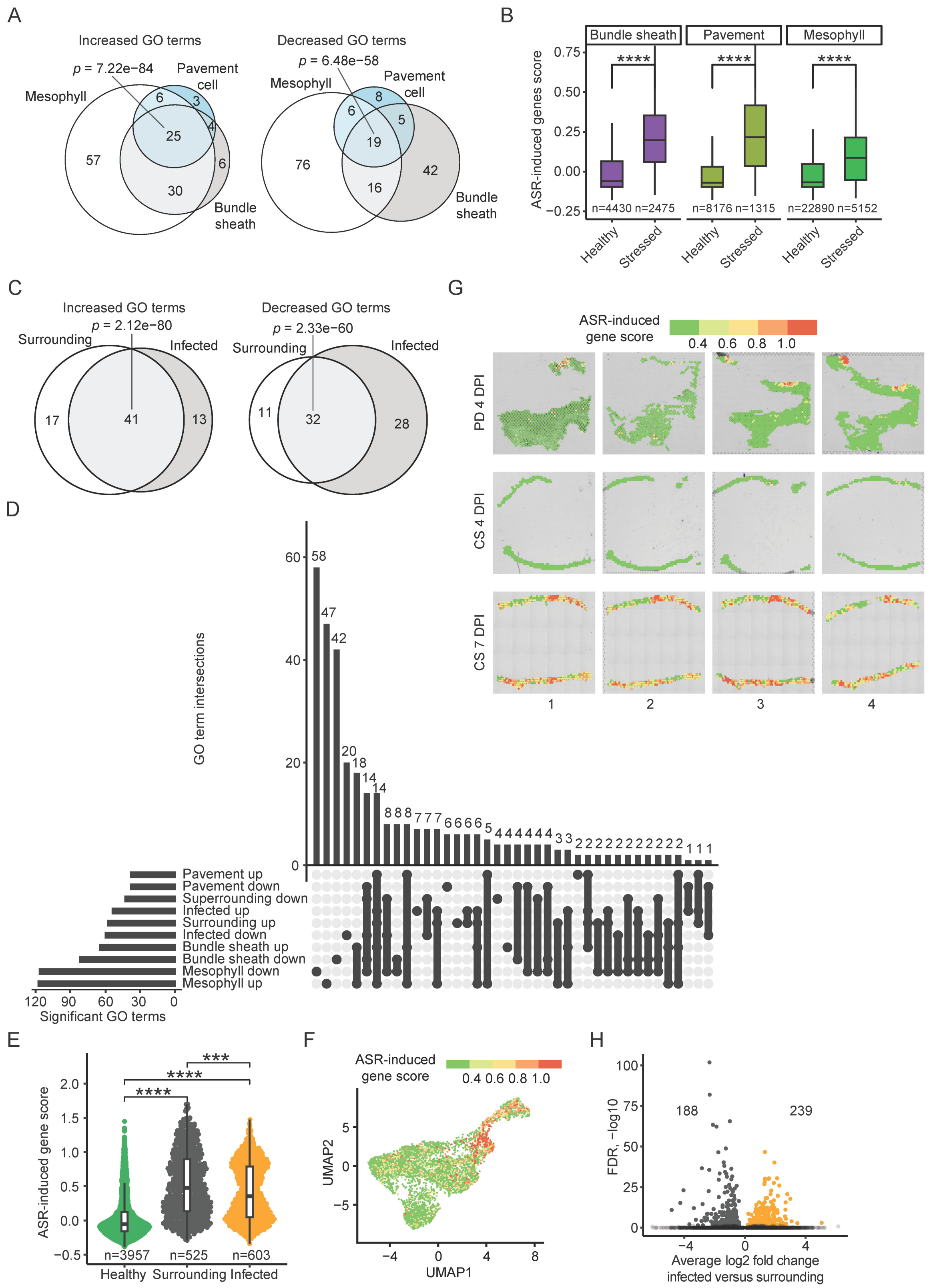
A
Increased GO terms
p = 7.22e−84
Mesophyll
Pavement cell
Bundle sheath
Decreased GO terms
p = 6.48e−58
B
Bundle sheath
Pavement
Mesophyll
ASR-induced genes score
Healthy
Stressed
C
Increased GO terms
p = 2.12e−80
Surrounding
Infected
Decreased GO terms
p = 2.33e−60
D
GO term intersections
Pavement up
Pavement down
Superrounding down
Infected up
Surrounding up
Infected down
Bundle sheath up
Bundle sheath down
Mesophyll down
Mesophyll up
Significant GO terms
E
ASR-induced gene score
n=3957
n=525
n=603
Healthy
Surrounding
Infected
F
ASR-induced gene score
UMAP1
UMAP2
G
ASR-induced gene score
PD 4 DPI
CS 4 DPI
CS 7 DPI
H
FDR, −log10
Average log2 fold change infected versus surrounding
188
239

Supplemental Figure 3. A) Venn diagrams showing agreement in significantly enriched and depleted GO terms in stressed cell types. Signficance determined by super exact test. **B)** ASR-induced gene expression described as a single score per nuclei in each cell type cluster or stressed subcluster. n indicates the number of nuclei for each category. * indicates $p < 0.05$, ** indicates $p > 0.01$, *** indicates $p < 0.001$, **** indicates $p < 0.0001$. **C)** Venn diagrams showing agreement in significantly enriched and depleted GO terms in surrounding and infected spatial regions compared to healthy regions, respectively. Significance determined by hypergeometric test. **D)** Upset plot comparing enriched GO terms for stressed versus healthy nuclei in each snRNA-seq cell type and for surrounding and infected spatial regions. **E)** ASR-induced gene expression described as a single score per spot in each spatial region. * indicates $p < 0.05$, ** indicates $p > 0.01$, *** indicates $p < 0.001$, **** indicates $p < 0.0001$. **F)** ASR-induced gene expression described as a single score across all spatial spots. **G)** ASR-induced gene expression described as a single score across all spots on all sections. **H)** Volcano plots of significantly differentially expressed soybean genes (padj < 0.05) in infected vs surrounding spatial regions.

Figure S4

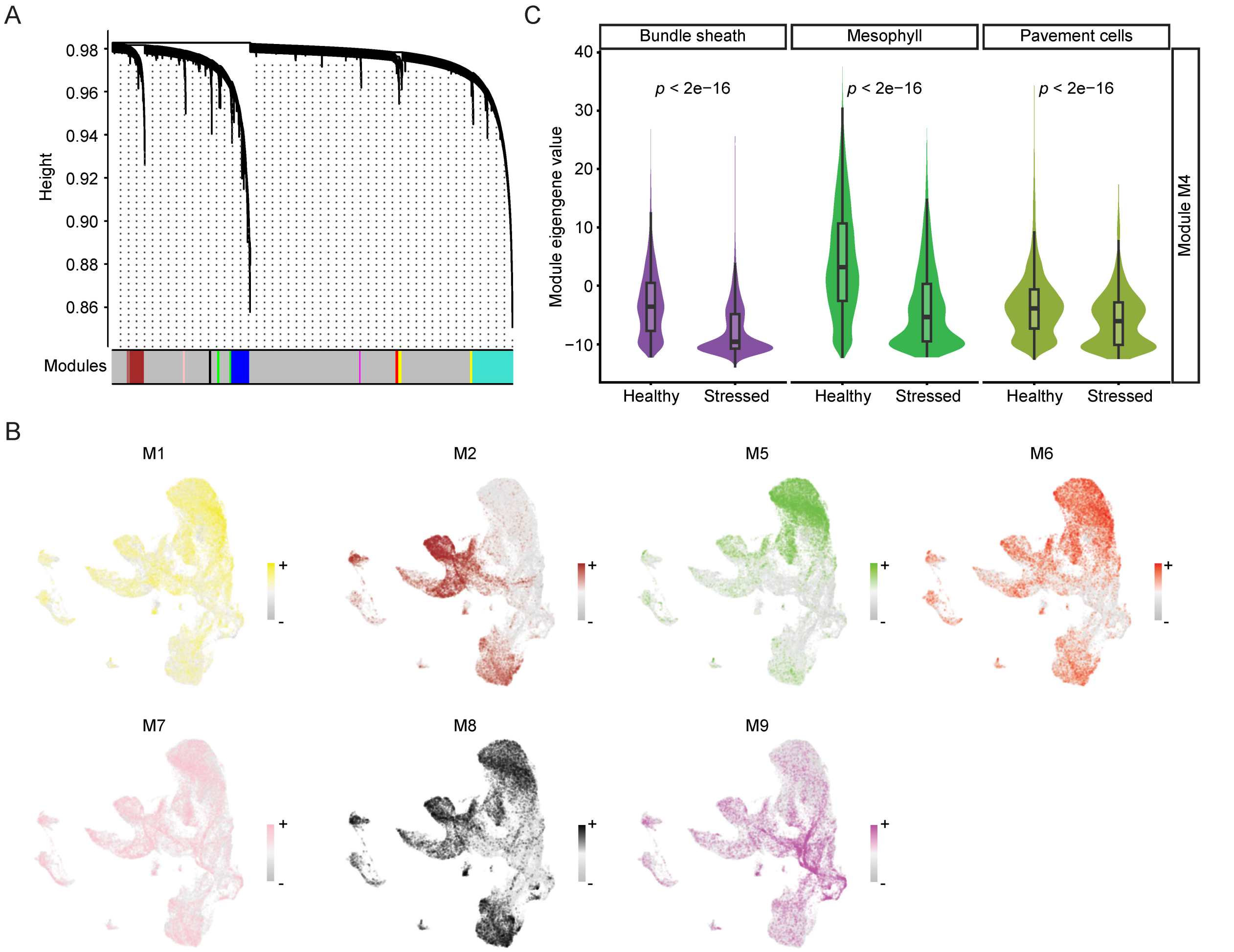

Supplemental Figure 4. A) Dendrogram of co-expression network modules constructed by hdWGCNA on mesophyll cells using snRNA-seq data. Colored bar indicates module assignment for each gene. **B)** Module eigengene values for M1, M2, and M5-9 plotted in snRNA-seq UMAP. **C)** Comparison of M4 module eigengene distributions in stressed and healthy cells across three cell types in snRNA-seq. Significance was determined via an unpaired two-sided Wilcoxon Rank Sum test.
